# Adjunctive extracorporeal shock wave therapy after botulinum toxin type A injection for post-stroke spasticity: a systematic review and three-level meta-analysis

**DOI:** 10.3389/fneur.2026.1890975

**Published:** 2026-10-20

**Authors:** Weihao Ke, Lujiang Wang, Wenli Shen, Min Chen, Zhenyu Wang

**Affiliations:** 1 Department of Critical Care Rehabilitation Medicine, Affiliated Rehabilitation Hospital of Chongqing Medical University, Chongqing, China; 2 Affiliated Rehabilitation Hospital of Chongqing Medical University, Chongqing, China

**Keywords:** activities of daily living, botulinum toxin type A, extracorporeal shock wave therapy, fugl-meyer assessment, modified ashworth scale, post-stroke spasticity, three-level meta-analysis

## Abstract

**Background:**

Post-stroke spasticity is a common rehabilitation problem, and botulinum toxin type A (BTX-A) is a standard focal treatment. Whether extracorporeal shock wave therapy (ESWT) provides clinically relevant additional benefit after BTX-A remains uncertain.

**Objective:**

This systematic review and three-level meta-analysis evaluated the add-on effect of ESWT after BTX-A for post-stroke spasticity, with particular attention to impairment-level and activities-of-daily-living outcomes. Methods: PubMed, Embase, Cochrane Library, Web of Science, CNKI, Wanfang Data, VIP, and CBM were searched from inception to March 2026. Randomized controlled trials of BTX-A plus ESWT in adult post-stroke spasticity were included. The primary quantitative synthesis was restricted to BTX-A-based comparators. Hedges' g was pooled with three-level random-effects models to account for dependent effect sizes.

**Results:**

Seven randomized controlled trials with 377 participants met the qualitative criteria, and six trials with 242 participants contributed to the primary quantitative synthesis. Adjunctive ESWT was associated with greater reduction in Modified Ashworth Scale scores (*g* = 0.87, 95% CI 0.41 to 1.34; *p* < 0.001) and greater improvement in Fugl-Meyer Assessment scores (*g* = 1.11, 95% CI 0.45 to 1.76; *p* < 0.001). Activities-of-daily-living estimates favored the combined intervention but did not reach conventional statistical significance (*g* = 1.15, 95% CI −0.03 to 2.32; *p* = 0.056). Two participants developed transient erythema after ESWT, but adverse-event reporting was too incomplete for a comparative safety estimate.

**Conclusion:**

Adjunctive ESWT may improve short-term impairment-level outcomes after BTX-A, but certainty is limited and evidence for reliable functional independence gains is very uncertain. Modality-, target-, timing-, and dose-specific findings remain exploratory.

**Systematic Review Registration:**

Identifier INPLASY202630010; https://doi.org/10.37766/inplasy2026.3.0010.

## Introduction

Stroke is a major cause of long-term disability worldwide. The global economic burden of stroke was estimated to exceed US$890 billion per year in 2021 and may double by 2050 (1). Post-stroke spasticity is common and clinically important, affecting 25% to 80% of survivors. Its frequency increases during the first year after stroke, from about 19% at 3 months to 38% at 12 months, and it can interfere with rehabilitation and activities of daily living (ADL) (2, 3). Botulinum toxin type A (BTX-A) is a standard focal treatment for spasticity (3, 4), but its effect is time-limited and may wane within 8 to 12 weeks (5). Its action is also mainly neural. It does not directly address non-neural contributors to hypertonia, such as muscle fibrosis, altered muscle architecture, and soft-tissue contracture (6).

Extracorporeal shock wave therapy (ESWT) has been studied as a non-invasive adjunct to BTX-A. Proposed mechanisms include changes at the neuromuscular junction and tissue-level mechanobiological responses, although these pathways have not been confirmed as mediators of clinical benefit in post-stroke spasticity (7, 8). Clinical studies have examined ESWT in both lower-limb (9) and upper-extremity spasticity (10), but the evidence is difficult to interpret because studies vary in ESWT modality, target muscle, outcome timing, and comparator structure. Previous meta-analyses have evaluated ESWT for post-stroke spasticity, but most assessed ESWT under sham, placebo, or conventional-control conditions rather than isolating the incremental effect of ESWT when added to BTX-A-based treatment (11–13). A recent ESWT-focused review addressed BTX-A combined with ESWT (14), and a network meta-analysis compared botulinum toxin combined with several rehabilitation strategies, including ESWT (15). These syntheses answer related but different questions: broad ESWT reviews do not isolate the add-on value of ESWT after injection, whereas network rankings of rehabilitation packages do not give a focused, outcome-domain-specific estimate for ESWT itself. A second problem is statistical. Neurorehabilitation trials commonly report several time points and related outcomes from the same participants. Standard two-level meta-analyses may either discard useful information or treat correlated effect sizes as independent, which can distort variance estimates and statistical inference (16, 17).

We therefore used a three-level random-effects meta-analysis to estimate the add-on effect of ESWT while accounting for repeated time points and related outcomes within trials. The quantitative synthesis was restricted to RCTs comparing ESWT plus BTX-A with BTX-A-based control conditions. We also used exploratory meta-regression to examine whether ESWT modality or treated limb location was associated with effect estimates. Because the number of independent study clusters was small, moderator findings were treated as exploratory rather than confirmatory.

## Methods

### Study protocol and registration

This systematic review followed the preferred reporting items for systematic reviews and meta-analyses (PRISMA 2020) guidelines (18). The protocol was registered prospectively in INPLASY (INPLASY202630010). The protocol is accessible at https://doi.org/10.37766/inplasy2026.3.0010.

The final analysis differed from the registered protocol in several respects. Quantitative synthesis was restricted to BTX-A-based comparators; trials without such comparators were retained for qualitative synthesis. The protocol specified selecting a fixed- or random-effects model according to statistical heterogeneity. We instead used three-level random-effects models with REML estimation to account for multiple outcomes and follow-up times within trials. Sensitivity analyses and exploratory meta-regressions were added, and GRADE was completed during revision. Safety findings were summarized narratively because reporting was incomplete.

### PICOS framework

The review question was defined according to the PICOS framework. The population was adults with post-stroke spasticity. The intervention was intramuscular BTX-A combined with focused or radial ESWT. Eligible comparators for the primary quantitative synthesis were BTX-A-based conditions, including BTX-A alone or BTX-A plus sham ESWT or conventional rehabilitation when BTX-A was present in the control arm. Outcomes included muscle tone measured by MAS, motor impairment measured by FMA, and activities of daily living measured by scales such as the Modified Barthel Index or Functional Independence Measure. Eligible study designs were randomized controlled trials. Safety outcomes comprised trial-reported adverse events and serious adverse events.

### Search strategy

We searched PubMed, Embase, the Cochrane Library, Web of Science, CNKI, Wanfang Data, VIP, and CBM from database inception to March 2026. The search combined medical subject headings (MeSH) and free-text terms for stroke, muscle spasticity, BTX-A, and ESWT. Database searches were not restricted by language. Reports in English or Chinese were eligible, as prespecified in the protocol.

### Selection criteria

Eligible studies enrolled adults (≥18 years) with post-stroke spasticity confirmed by clinical assessment and neuroimaging when available. The intervention of interest was intramuscular BTX-A combined with focused or radial ESWT. For the primary quantitative synthesis, the comparator had to include a BTX-A-based baseline condition, either BTX-A alone or BTX-A plus sham ESWT. Trials that compared the combined intervention only with rehabilitation or other non-BTX-A control conditions were retained for qualitative consideration when relevant but were not included in the primary add-on effect model. Eligible outcomes included the modified ashworth scale (MAS), the Fugl-Meyer assessment (FMA), and ADL scales. Only randomized controlled trials in human participants were eligible.

Studies were excluded if they did not provide a clear post-stroke diagnosis, lacked usable outcome data, included spasticity caused by non-stroke conditions, or used co-interventions that made the add-on effect of ESWT impossible to isolate. Non-randomized studies, reviews, conference abstracts, case reports, and protocols without primary empirical data were also excluded.

### Study selection

Records were screened by title and abstract, followed by full-text assessment against the eligibility criteria.

### Data extraction and MAS transformation

Two investigators independently extracted data with a standardized electronic form. Disagreements were resolved by discussion or by consultation with a senior author. For MAS, ordinal grades were converted to a 6-point numeric scale to allow quantitative synthesis: Grade 0 = 0, Grade 1 = 1, Grade 1+ = 1.5, Grade 2 = 2, Grade 3 = 3, and Grade 4 = 4. Reported adverse events were extracted and summarized narratively because definitions and ascertainment differed across trials and reporting was incomplete.

### Quality assessment

Risk of bias was assessed independently with the revised Cochrane Risk of Bias tool (RoB 2.0) (19). The assessment covered randomization, deviations from intended interventions, missing outcome data, outcome measurement, and selective reporting.

### Certainty of evidence

Certainty of evidence for the three prespecified core outcome domains (MAS, FMA, and ADL) was assessed using the GRADE approach (20). Randomized-trial evidence was initially rated as high certainty and was then considered for downgrading across five domains: risk of bias, inconsistency, indirectness, imprecision, and publication bias. Judgments were made at the outcome level and were based on the number of independent trials rather than the number of dependent effect sizes. The width and clinical implications of the 95% confidence interval, study-level heterogeneity, protocol diversity, and the outcome-specific RoB 2 profile were considered. Because fewer than 10 independent studies contributed to each outcome, formal funnel-plot asymmetry or regression-based tests were not used to support publication-bias judgments. A Summary of Findings table presents the final certainty ratings and reasons for downgrading (Table 1).

**Table 1:** GRADE summary of findings.

Outcome	Trials/participants	Pooled effect, Hedges g (95% CI)	GRADE certainty	Main reasons for rating	Interpretation
MAS/spasticity	6 RCTs/242	0.87 (0.41 to 1.34)	Moderate	Downgraded for imprecision: small independent trial base and uncertainty in the magnitude of the standardized effect.	Adjunctive ESWT probably reduces muscle tone more than BTX-A-based control, but the magnitude remains uncertain.
FMA/motor impairment	5 RCTs/202	1.11 (0.45 to 1.76)	Low	Downgraded for inconsistency and imprecision: substantial between-study variance, protocol diversity, and a wide standardized-effect interval.	Adjunctive ESWT may improve limb-level motor impairment, but confidence in the magnitude is limited.
ADL/functional independence	6 RCTs/242	1.15 (-0.03 to 2.32)	Very low	Downgraded for risk of bias, inconsistency, and imprecision: one contributing trial had high risk in outcome measurement; the influential focused-ESWT trial had marked baseline imbalance; between-study heterogeneity was dominant; and the CI crossed the null.	Whether adjunctive ESWT improves global functional independence is very uncertain.

GRADE note: randomized-trial evidence starts at high certainty. No separate downgrade was applied solely because formal publication-bias tests were unavailable; however, publication bias cannot be excluded. Hedges g is a standardized effect and does not map directly to a single established minimal clinically important difference across the different MAS, FMA, and ADL instruments and follow-up times.

### Statistical analysis

Analyses were conducted in R. The initial models used R v4.3.1 and metafor v4.4-0; the revised FMA and CR2 analyses used R v4.6.0, metafor v5.0-1, and clubSandwich v0.7.0. The primary estimand was the additional effect of ESWT when combined with BTX-A, compared with BTX-A-based control conditions. Effect sizes were calculated as Hedges' g and coded so that positive values favored ESWT plus BTX-A. The primary models used post-treatment standardized mean differences because this metric could be extracted consistently across eligible trials and follow-up points. Three-level random-effects meta-analysis was used to account for statistical dependence arising from repeated follow-up assessments, multiple treated muscles, and related outcomes within the same trial (16, 17). The model partitioned total variance into Level 1 sampling variance, Level 2 within-study variance, and Level 3 between-study heterogeneity.

Model-based estimates and 95% confidence intervals are reported in the main analyses and forest plots. Small-sample robustness was examined with the bias-reduced cluster-robust variance estimator (CR2), clustered by study and using Satterthwaite degrees of freedom. Multilevel models were fitted by restricted maximum likelihood (REML) with rma.mv (21). Clinical comparability was handled separately by limiting the primary quantitative synthesis to trials with BTX-A-based comparator arms. Sensitivity analyses tested whether findings changed after excluding Ren et al. (22), which had high risk in the measurement of outcomes domain; restricting the analysis to the follow-up closest to week 4; aggregating effect sizes at the study level for the closest week-4 follow-up; excluding the single focused-ESWT trial (10) for ADL; and excluding ADL effect sizes with major baseline imbalance. Because ADL outcomes were particularly sensitive to baseline differences, change-score SMD models were also calculated under assumed pre-post correlations of 0.2, 0.5, and 0.8, following standard methods for continuous outcomes in meta-analysis (23). Univariate exploratory meta-regressions examined ESWT modality, limb location, follow-up period, time point, number of ESWT sessions, interval from BTX-A to ESWT, and stroke stage. These moderator analyses were considered exploratory because the number of independent study clusters was small.

## Results

### Study selection and characteristics

The search identified 72 records across eight databases (PubMed, *n* = 8; Embase, *n* = 16; Cochrane Library, *n* = 9; Web of Science, *n* = 10; CNKI, *n* = 11; Wanfang Data, *n* = 8; CBM, *n* = 7; VIP, *n* = 3). After 27 duplicates were removed, 45 records were screened and 13 reports were sought for retrieval. Five reports could not be retrieved, leaving eight reports for eligibility assessment. One report was excluded before qualitative synthesis because it did not provide an eligible primary empirical comparison for the review question. Seven RCTs contributed 377 participants to the qualitative review. Six trials and 242 participants contributed to eligible BTX-A-based comparisons (Figure 1). The other 135 participants were from Liang et al. (24) and non-BTX-A arms in three multi-arm trials; the denominator breakdown is provided in the Supplementary eTables 3.

**Figure 1:**
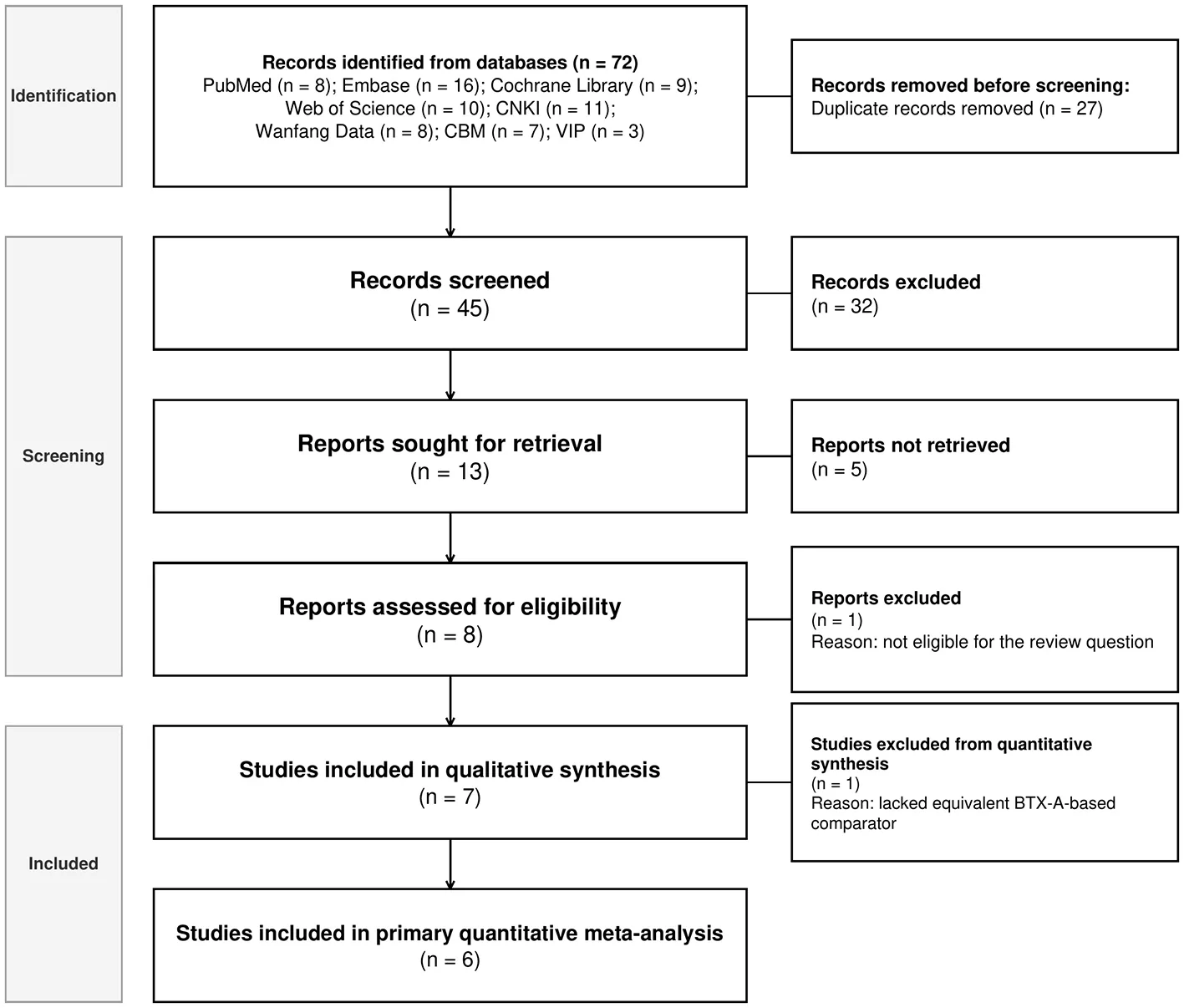
PRISMA 2020 flow diagram. The search identified 72 records across eight databases. After 27 duplicate records were removed, 45 records were screened. Thirteen reports were sought for retrieval, eight reports underwent eligibility assessment, and seven RCTs were included in the qualitative synthesis. One trial without an equivalent BTX-A-based comparator was excluded from the primary quantitative synthesis, leaving six RCTs for the three-level meta-analysis.

Trial characteristics and intervention protocols are provided in the Supplementary eTables 3, 4.

Intervention protocols varied substantially across trials (Table 2). Five of the six trials in the primary quantitative synthesis used radial ESWT, whereas Lee and Yang (10) used focused ESWT. Radial protocols used pressures of approximately 1.8–3.0 bar, with 1,000–2,000 impulses per session in most trials; the focused protocol used an energy flux density of 0.030 mJ/mm^2^ with 1,000 shocks per session. Total ESWT exposure ranged from 4 to 24 sessions, and the first ESWT session occurred from immediately after BTX-A injection to 7 days later. Anatomical targets also differed: upper-limb trials treated elbow, wrist, and/or finger flexor patterns, whereas lower-limb trials primarily targeted the gastrocnemius, soleus, triceps surae, or ankle plantar-flexor pattern. Ren and Hu (22) explicitly enrolled patients with unilateral spasticity affecting both the upper and lower limbs. BTX-A protocols were similarly heterogeneous, ranging from fixed or muscle-specific dosing to individualized multi-muscle injection, and several reports provided a reconstitution concentration without a comparable total dose. These differences precluded a meaningful cross-trial analysis of a BTX-A dose-ESWT interaction. BI and MBI are global measures of functional independence rather than upper- or lower-limb-specific scales; limb-specific information therefore refers to the treated musculature and impairment-level outcomes such as MAS and FMA.

**Table 2:** Clinical and intervention characteristics of included trials.

Study	Use/N	Target/stage	BTX-A protocol	ESWT protocol	Sessions/timing	Outcome scope
Talay Çaliş et al. (25)	Quantitative; 40 (20/20)	Lower; chronic ankle plantar-flexor spasticity	US-guided gastrocnemius; medial head 50 IU + lateral head 25 IU	Radial; medial/lateral gastrocnemius; 0.1 mJ/mm^2^ and 2 bar; 4 Hz; 1,000 impulses	4 sessions (2/week); starts day 7	MAS + global BI
Deng (32)	Quantitative; 50 in add-on contrast	Upper; elbow/wrist/finger flexor spasticity	US-guided individualized target muscles; Botox 100 U/2 mL preparation; exact total dose and named muscles not reported	Radial; individualized spastic target-muscle bellies; 1.8–2.5 bar; 8–10 Hz; 2,000 impulses	24 sessions (2/week x12 weeks); starts day 3 (~48 h)	MAS; upper-extremity FMA; global MBI
Duan et al. (9)	Quantitative; 36 (18/18)	Lower; subacute triceps surae	Electrical-stimulation guidance; gastrocnemius medial/lateral heads + soleus; individualized; maximum 100 U per muscle group	Radial; triceps surae; 2.0–3.0 bar; 8 Hz; 2,000 impulses	Regimen: daily on post-injection days 1–3, then every 3 days from week 2 through week 4; starts 24 h after BTX-A	MAS; lower-extremity FMA; global MBI
Lee and Yang (10)	Quantitative; 16 (9/7)	Upper; chronic elbow/wrist/finger flexors	Electrical-stimulation guidance; individualized biceps brachii, brachioradialis, pronator teres, FCR, FCU, FDP, FDS; muscle-specific doses reported	Focused; middle belly of biceps brachii or FCR; EFD 0.030 mJ/mm^2^; 4 Hz; 1,000 shots	5 daily sessions; begins immediately	MAS/MTS; upper-extremity FMA; ARAT; global MBI
Ren and Hu (22)	Quantitative; 60 in add-on contrast	Both upper and lower limbs; subacute unilateral spasticity	US-guided individualized targets; 2–4 sites/muscle, 10–25 U/site; maximum < =600 U per treated side; exact named targets not listed	Radial; target muscle belly; 2 bar; 6 Hz; EFD 0.03–0.24 mJ/mm^2^; 2,000 impulses/muscle group	4 weekly sessions; starts day 3 (~48 h)	Affected-limb MAS; total FMA (UE+LE); global MBI
Wang et al. (34)	Quantitative; 40 in add-on contrast	Lower; chronic ankle plantar-flexor/triceps surae pattern	EMG guidance; candidates included gastrocnemius, soleus, tibialis posterior, FHL, FDL; individualized; total 400 U	Radial; gastrocnemius + soleus; 2 bar; 8 Hz; 1,500–2,000 impulses	4 weekly sessions; starts next day (~24 h)	MAS of ankle plantar flexors; lower-extremity FMA; global MBI
Liang et al. (24)	Qualitative only; 60	Upper limb	US-guided individualized visibly spastic target muscles; Botox 100 U/2 mL preparation; exact named muscles/total dose not reported	Modality not explicitly labeled in the available report; shock wave applied to the spastic muscle belly; 2 bar; 10 Hz; 2,000–3,000 impulses	12 sessions (2/week × 6 weeks); starts day 2	Upper-limb MAS/FMA; global MBI

Exact muscles and dose values are reported only when explicitly stated in the primary article. A reconstitution concentration is not treated as a total injected dose. BI and MBI are global ADL measures rather than limb-specific functional tests. Liang et al. (24) was excluded from the quantitative add-on model because its comparator lacked a BTX-A baseline.

Safety reporting was incomplete. Talay Çaliş et al. (25) reported transient erythema in 2 of 20 participants receiving adjunctive ESWT; symptoms resolved after approximately 20 min and no other side effects were reported. Duan et al. reported no obvious adverse reactions, and Lee and Yang (10) reported no serious adverse effects or complications. Liang et al. (24) reported no allergic reactions after BTX-A but did not otherwise summarize ESWT-related adverse events. The remaining three trials did not provide usable trial-level safety data. The data did not permit a comparative safety estimate.

### Methodological quality

Risk of bias was appraised using the RoB 2.0 tool (19). Among the six trials contributing to the primary quantitative synthesis, four were rated at low overall risk, Duan et al. (9) was rated as having some concerns, and Ren et al. (22) had high overall risk because of the measurement of outcomes domain (Supplementary eFigures 1, 2).

### Variance partitioning and model validation

Variance partitioning showed that the three-level structure was appropriate for these data (Figure 2). For MAS, variance was concentrated mainly within studies: Level 1 sampling error accounted for 20.2%, Level 2 within-study variance for 61.9%, and Level 3 between-study heterogeneity for 17.9%. For FMA, the corresponding values were 17.9%, 17.8%, and 64.3%. For ADL, most variance was between studies (90.7%), with 9.3% attributed to sampling error and no meaningful Level 2 component.

**Figure 2:**
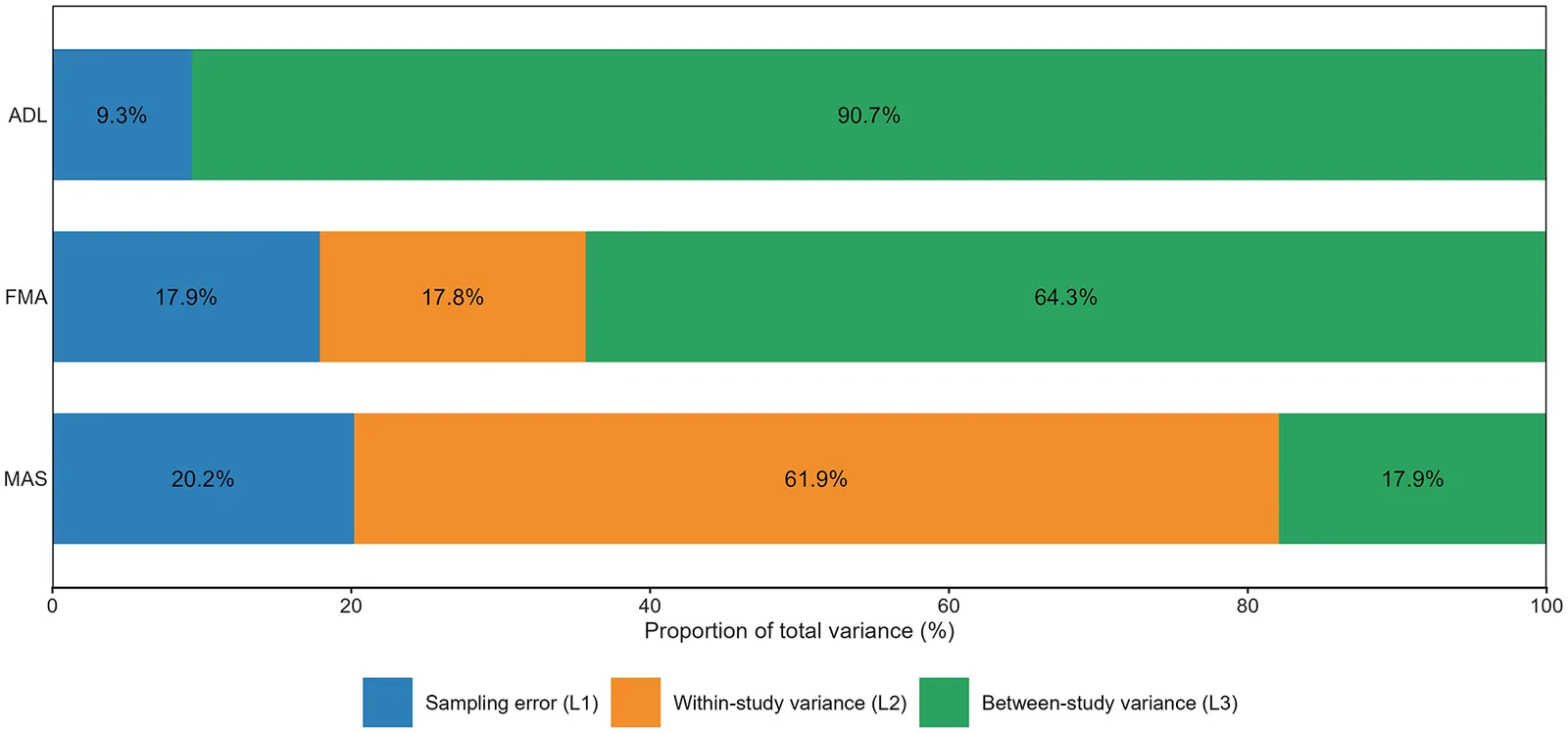
Variance decomposition from three-level meta-analytic models. Values show the percentage of total heterogeneity attributed to Level 1 sampling error, Level 2 within-study variance, and Level 3 between-study heterogeneity for MAS, FMA, and ADL outcomes.

### Primary outcome: muscle spasticity

The MAS analysis included 21 effect sizes from six trials with BTX-A-based control conditions (*N* = 242). The model-based estimate favored ESWT plus BTX-A (Hedges' *g* = 0.87; 95% CI: 0.41 to 1.34; *p* < 0.001; Figure 3A). The CR2 sensitivity interval was 0.25 to 1.49 (*p* = 0.016).

**Figure 3:**
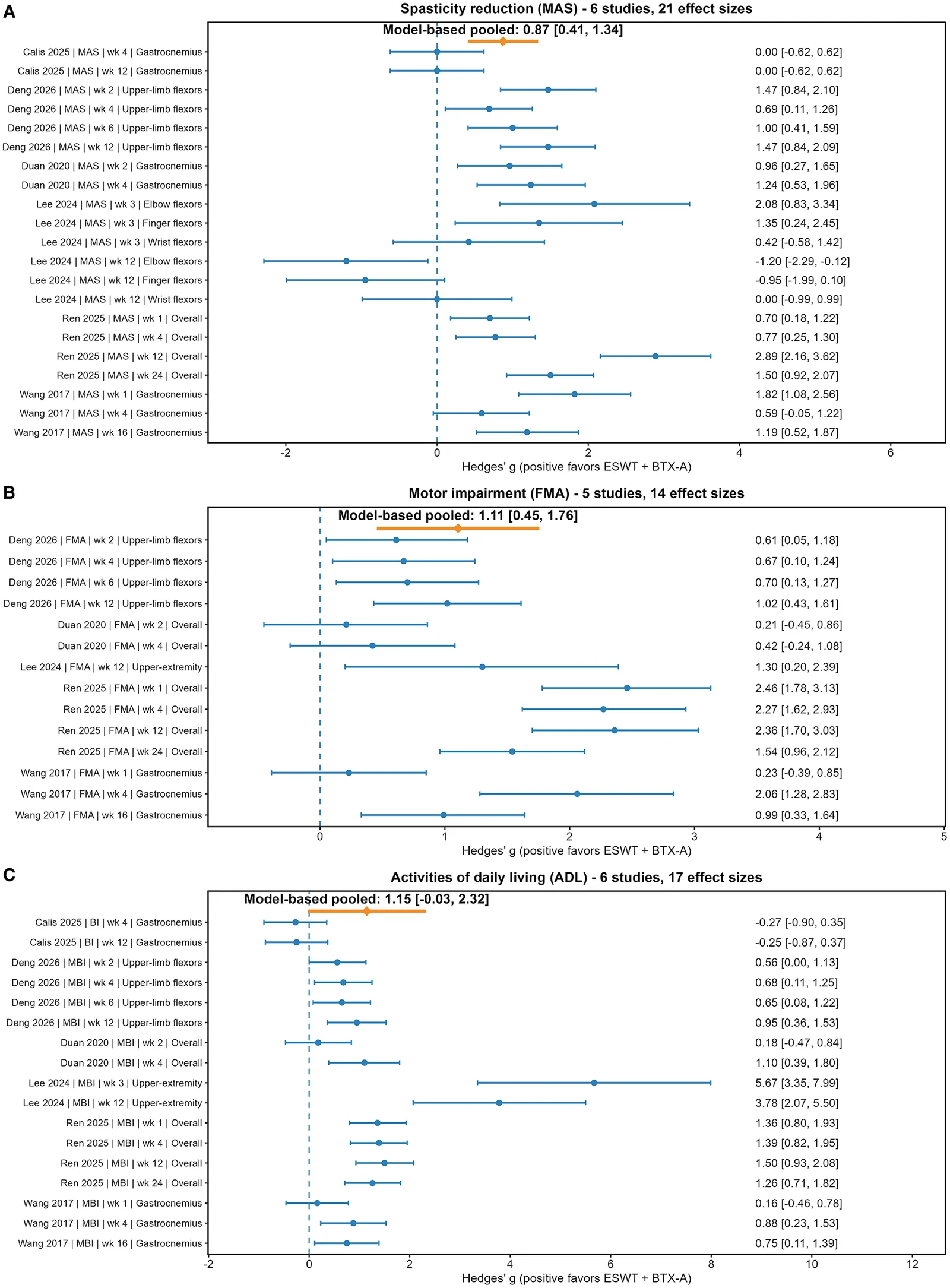
Forest plots comparing ESWT plus BTX-A with BTX-A-based control conditions. Panel **(A)** shows Modified Ashworth Scale (MAS) outcomes, Panel **(B)** shows Fugl-Meyer Assessment (FMA) outcomes, and Panel **(C)** shows activities of daily living (ADL) outcomes. Effect sizes are reported as Hedges' g with model-based 95% confidence intervals; CR2 sensitivity results are reported in Supplementary eTable 7.

### Secondary outcomes

#### Fugl-Meyer Assessment

The FMA analysis included 14 post-treatment effect sizes from five trials. The model-based estimate favored the combined intervention (*g* = 1.11; 95% CI: 0.45 to 1.76; *p* < 0.001; Figure 3B). The CR2 interval was wider but remained above zero (0.16 to 2.05; *p* = 0.032).

#### Activities of daily living

ADL was assessed with 17 effect sizes from six trials. The model-based estimate favored ESWT plus BTX-A, but the confidence interval crossed the null (*g* = 1.15; 95% CI: **–** 0.03 to 2.32; *p* = 0.056; Figure 3C). The CR2 interval was **–** 0.39 to 2.68 (*p* = 0.112).

### Exploratory meta-regression and subgroup analysis

Univariate meta-regressions examined clinical and intervention moderators (Supplementary eTable 6). For MAS, ESWT modality approached conventional statistical significance (QM = 3.80, *p* = 0.051), and stroke stage showed an exploratory study-level association with effect-size estimates (QM = 4.53, *p* = 0.033). Limb location, follow-up period, assessment time point, ESWT session number, and timing after BTX-A were not significant moderators. For FMA, none of the six retained moderators reached statistical significance.

For ADL, ESWT modality was associated with effect-size estimates in the post-treatment SMD model (QM = 16.94, *p* < 0.001). This result was driven by the single focused-ESWT trial and should not be read as confirmatory evidence that one ESWT modality is superior to another. The radial-ESWT subgroup had a smaller positive estimate (*g* = 0.62; 95% CI: 0.11 to 1.13), whereas the focused-ESWT estimate remained hypothesis-generating (Figure 4A). Limb location was not a significant ADL moderator in the primary model (QM = 2.28, *p* = 0.131) or after exclusion of the focused-ESWT trial (QM = 0.53, *p* = 0.468). Stroke stage was associated with ADL estimates in exploratory meta-regression (QM = 7.59, *p* = 0.006), but this signal was not treated as confirmatory because it was based on few study-level clusters and could reflect between-study confounding.

**Figure 4:**
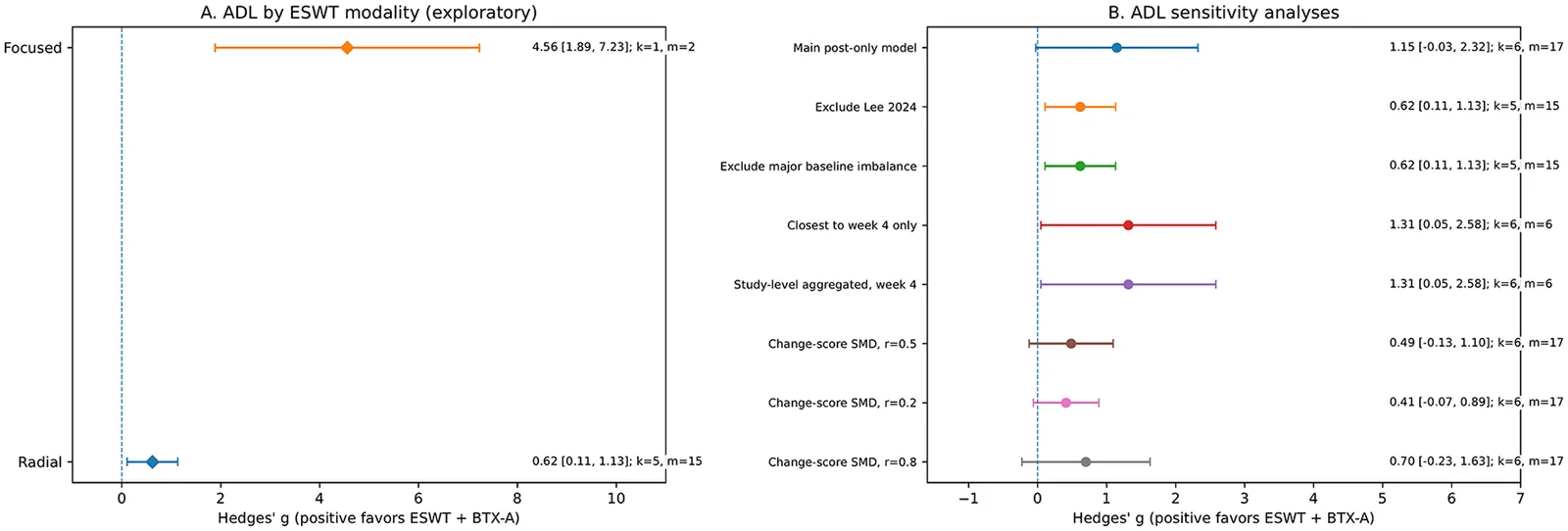
Exploratory ADL moderator and sensitivity analyses. Panel **(A)** shows ADL effect estimates stratified by ESWT modality. The focused-ESWT estimate came from a single small trial and is sensitive to baseline imbalance; it should be interpreted as hypothesis-generating. Panel **(B)** summarizes ADL sensitivity analyses, including study exclusions, closest-week-4 models, study-level aggregation, and change-score SMD models under different assumed pre-post correlations.

### Sensitivity analysis and publication bias

Sensitivity analyses supported the direction of the MAS and FMA findings (Supplementary eTables 5, 7). In model-based leave-one-out analyses, all estimates remained positive and statistically significant (MAS, *g* = 0.74 to 1.03; FMA, *g* = 0.77 to 1.31). With CR2, all FMA point estimates remained positive, although intervals crossed the null after omitting Deng, Lee, or Wang (Figure 5). The closest-week-4 FMA estimate was 1.33 (95% CI: 0.58 to 2.08; *p* < 0.001), with a CR2 interval of 0.26 to 2.41 (*p* = 0.026). ADL was less stable. The main post-treatment model did not reach conventional statistical significance (*g* = 1.15; 95% CI: **–**0.03 to 2.32; *p* = 0.056). Excluding Lee et al. (10), or excluding effect sizes with major baseline imbalance, yielded a smaller positive estimate (*g* = 0.62; 95% CI: 0.11 to 1.13; *p* = 0.018). However, change-score SMD sensitivity analyses under assumed pre-post correlations of 0.2, 0.5, and 0.8 all produced confidence intervals crossing the null (Figure 4B). Because fewer than 10 independent trials contributed to each outcome, formal funnel-plot asymmetry tests and regression-based small-study-effect tests were not performed. Publication bias therefore cannot be ruled out. Qualitatively, the evidence base is small and largely composed of modest-sized single-center trials, which increases vulnerability to selective publication and selective reporting. We therefore make no directional claim about publication bias and treat the inability to assess it formally as an additional limitation.

**Figure 5:**
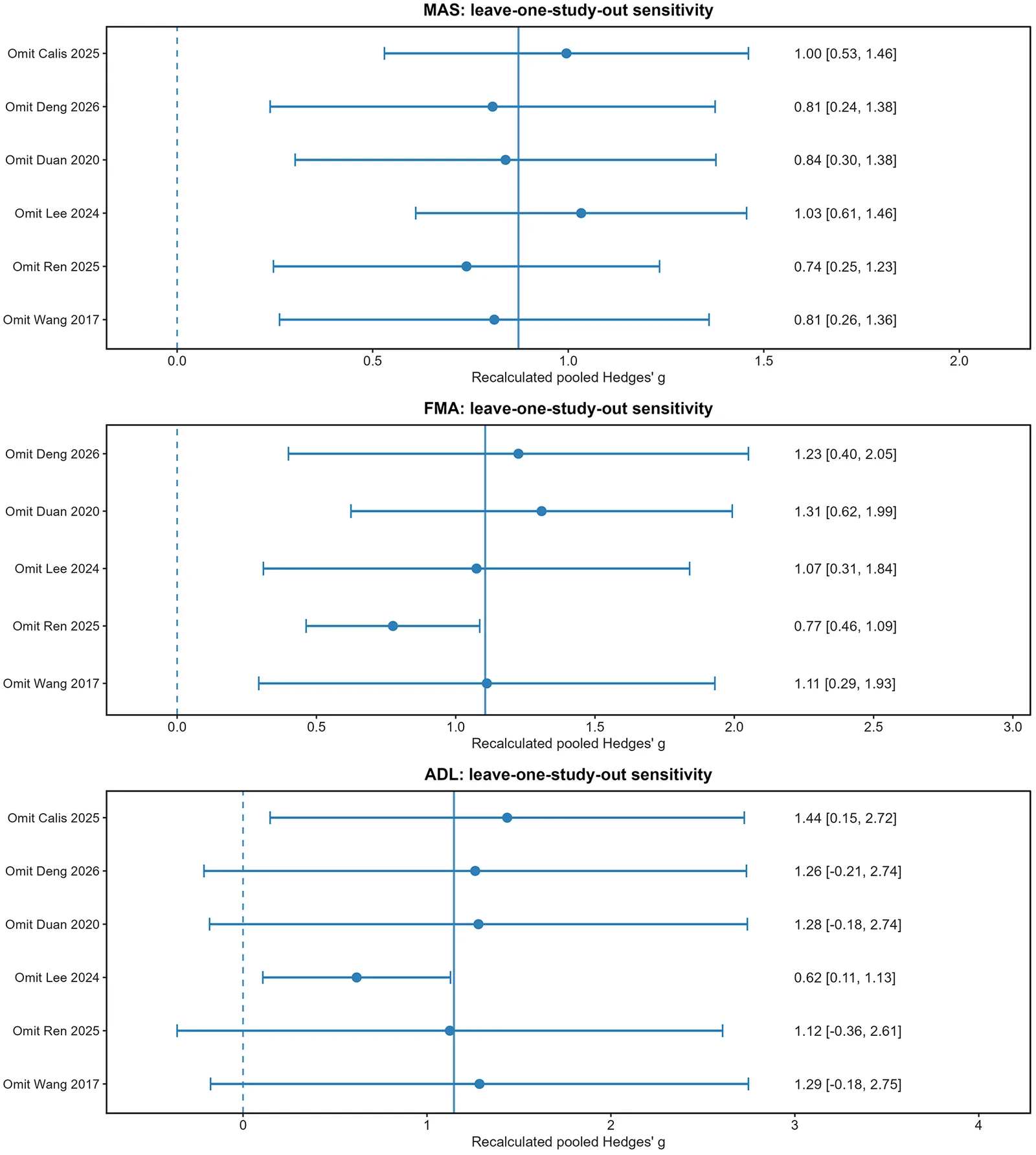
Leave-one-out sensitivity analyses for MAS, FMA, and ADL outcomes. Each estimate represents the model-based pooled effect after one study was omitted. All FMA point estimates remained positive; CR2 intervals crossed the null after omission of Deng, Lee, or Wang (Supplementary eTable 7). ADL estimates were more sensitive to study composition.

### Certainty of evidence

The certainty of evidence was rated as moderate for MAS, low for FMA, and very low for ADL (Table 1). For MAS, certainty was downgraded for imprecision because the independent trial base was small and the standardized-effect interval left uncertainty about the magnitude of benefit. For FMA, certainty was downgraded for inconsistency and imprecision, with substantial between-study variance and protocol diversity despite a pooled estimate favoring the combined intervention. For ADL, certainty was downgraded for risk of bias, inconsistency, and imprecision: one contributing trial had high risk in the measurement of outcomes domain; the influential focused-ESWT trial also had marked baseline imbalance relevant to post-treatment SMDs; between-study heterogeneity dominated the variance structure; the primary 95% CI crossed the null; and change-score sensitivity models under all assumed pre-post correlations also crossed the null. Publication bias could not be formally assessed for any outcome because fewer than 10 independent studies were available; this limitation was not interpreted as evidence that publication bias was absent.

## Discussion

### Principal findings and evidence strength

This review addressed a focused clinical question that is directly relevant to post-stroke spasticity management: whether ESWT provides incremental benefit when added to BTX-A-based treatment, and whether that benefit extends beyond impairment-level outcomes. Compared with studies using conventional rehabilitation as the control condition, this comparator framework may better clarify the added value of ESWT beyond the known therapeutic effect of BTX-A (3, 4). The main finding is not simply that adjunctive ESWT may reduce spasticity. Rather, the evidence was most consistent for impairment-level outcomes. ESWT plus BTX-A was associated with greater reductions in MAS scores and greater improvements in FMA scores, and the direction of both estimates was preserved across sensitivity analyses.

These findings should be read alongside previous syntheses rather than as a replacement for them. Du et al. (14) reviewed BTX-A combined with ESWT for post-stroke spasticity, and Ke et al. (15) ranked botulinum toxin combined with several rehabilitation strategies in a network meta-analysis. The present review complements those studies by narrowing the comparator to BTX-A-based control conditions, focusing on ESWT as a specific adjunct, separating impairment-level effects from ADL-level uncertainty, and retaining dependent effect sizes through a three-level model. This narrower design is useful for the clinical question faced after BTX-A injection: whether adding ESWT is likely to provide benefit beyond injection-based care alone.

Evidence for ADL improvement was less consistent. The primary analysis favored the combined intervention, but the confidence interval crossed the null. The apparent ADL signal was also sensitive to modeling choices and to one small focused-ESWT trial with baseline imbalance. In change-score sensitivity analyses, the ADL confidence intervals crossed the null under all assumed pre-post correlations. The most cautious interpretation is that adjunctive ESWT may improve spasticity and motor impairment, but current evidence does not yet show that these gains reliably translate into better functional independence.

This distinction is clinically important. Although reductions in spasticity and improvements in limb-level motor performance are meaningful therapeutic outcomes, they do not necessarily translate into greater independence in self-care or mobility. The international classification of functioning, disability and health (ICF) distinguishes impairments in body function from activity limitations and participation restrictions (26). The present findings are consistent with this framework. ESWT may improve the neuromuscular conditions necessary for rehabilitation practice; however, improvements in activities of daily living typically depend on additional factors, including task-specific training, sufficient practice intensity, environmental support, and time for motor relearning and functional adaptation (27).

### Clinical interpretation: from tone reduction to function

BTX-A is an established focal treatment for post-stroke spasticity, but its clinical effect is limited by finite duration of action, dose-related constraints, and non-neural contributors to hypertonia, including soft-tissue stiffness, contracture, and structural muscle changes (3–6). ESWT may help by acting on tissues and neuromuscular structures that are not fully addressed by chemodenervation, including potential effects on neuromuscular junction integrity and soft-tissue remodeling (7, 8, 28, 29). The MAS and FMA findings are consistent with this interpretation. In practical terms, the pooled results suggest that ESWT may create more favorable biomechanical conditions for active rehabilitation after injection, but the certainty assessment does not support treating this interpretation as a routine clinical recommendation.

The ADL findings should not be interpreted as absence of clinical benefit. ADL is a downstream outcome within the activity domain rather than a direct measure of body-function impairment (26). A focal reduction in gastrocnemius, wrist-flexor, finger-flexor, or elbow-flexor tone may make movement practice easier, but it does not by itself retrain walking, dressing, transfers, or self-care. Improvements in these activities generally require task-specific, goal-directed, and repetitive practice (27). ADL scales also combine many domains, and their sensitivity depends on baseline disability, rehabilitation exposure, follow-up duration, and the specific functional tasks affected by the treated muscles. This helps explain why impairment-level outcomes were more consistent than ADL outcomes.

Future trials should define the intended treatment target more clearly. If the aim is spasticity reduction, MAS and related tone measures are appropriate primary outcomes. If the aim is functional independence, trials should combine injection-based treatment with a standardized task-specific rehabilitation program (27), prespecify functional endpoints, and report both endpoint and change-score data. Without this design, a trial may demonstrate better tone control while leaving the functional meaning of that improvement uncertain.

### ESWT modality, anatomical target, and protocol dose

ESWT modality was associated with ADL effect estimates in exploratory meta-regression, but this result should not be overread. The large focused-ESWT estimate came from one small trial that also contributed the main baseline-imbalance concern for ADL. The finding may reflect a true modality-related difference, but it may also reflect study-specific factors such as sample size, baseline functional status, stroke chronicity, outcome scale, rehabilitation exposure, or follow-up timing. The available evidence cannot distinguish these explanations.

The radial-ESWT subgroup showed a smaller positive ADL estimate after the focused-ESWT trial was excluded. This suggests that the overall ADL signal was not explained only by the extreme focused-ESWT estimate. It does not show that radial ESWT is a definitive standard, nor does it show that focused ESWT is superior. At present, the safer conclusion is that radial ESWT has broader clinical evidence in this dataset, whereas focused ESWT remains insufficiently studied in BTX-A-based add-on trials.

The limb-location analysis also requires restraint. Limb location was not statistically associated with ADL effects, and the result remained non-significant after the focused-ESWT trial was removed. This does not show anatomical equivalence. Testing equivalence would require a prespecified margin and a study design powered for that purpose (30). The available data only show that limb location did not emerge as a reliable moderator in this small dataset.

The protocol data also argue against inferring a single optimal ESWT regimen from this review. Radial pressure, focused-ESWT energy flux density, impulse number, treatment frequency, session number, injection-to-ESWT interval, anatomical target, and BTX-A dosing were not standardized. The exploratory meta-regressions did not identify associations of total ESWT sessions or injection-to-ESWT timing with MAS or FMA estimates. The apparent ADL association with ESWT modality was driven by one small focused-ESWT trial with baseline imbalance and is therefore not evidence that focused ESWT is superior. A recent broader meta-analysis of ESWT vs. sham or no intervention reported a study-level dose-response relationship for MAS (31), but that analysis addressed a different comparator structure and cannot establish a BTX-A dose-ESWT interaction in the present add-on dataset. Establishing such an interaction would require substantially more trials with standardized toxin-dose reporting or individual-participant data.

### Biological rationale and limits of mechanistic inference

There is a plausible biological rationale for combining BTX-A and ESWT. BTX-A reduces neuromuscular transmission mainly by inhibiting presynaptic acetylcholine release at the neuromuscular junction (5). ESWT has been proposed to affect spasticity through peripheral tissue and neuromuscular mechanisms, including changes in neuromuscular junction integrity, soft-tissue properties, and mechanotransduction (7, 8, 28, 29). These processes could complement chemodenervation by addressing both neural and non-neural contributors to post-stroke hypertonia (6).

The present meta-analysis, however, does not prove any of these mechanisms. The included clinical trials were not designed as mediation studies, and most did not measure muscle architecture, elastography, perfusion, electrophysiology, neuromuscular junction morphology, or molecular markers. The cellular findings in one included study provide indirect biological support, but they do not prove that the pooled clinical effects were mediated by mitochondrial autophagy or any other specific intracellular pathway (32).

Mechanistic claims should therefore remain limited. The clinical evidence supports an add-on association for impairment-level outcomes, not a confirmed biological pathway. Future mechanistic trials should combine standardized clinical endpoints with objective biomarkers or imaging-based measures, so that investigators can test whether changes in tone are mediated by tissue stiffness, neuromuscular transmission, perfusion, or cellular repair pathways.

### Methodological contribution

A methodological strength of this review is that it retained the dependent structure of the evidence rather than reducing each trial to a single selected endpoint. Neurorehabilitation trials often report several follow-up times, treated muscles, and related outcome domains from the same participant cohort. A conventional two-level meta-analysis would either discard clinically relevant information or treat correlated estimates as independent. The three-level model allowed us to retain these data while separating sampling error, within-study variance, and between-study heterogeneity (16, 17).

The variance decomposition supports the use of this approach. MAS variance was concentrated within studies, which is consistent with repeated follow-up assessments and related treated targets. FMA and ADL had larger between-study components. This was especially clear for ADL, where between-study variance accounted for about 90.7% of the total variance. This pattern suggests that ADL effects were shaped mainly by study-level differences in participants, intervention protocols, outcome instruments, and follow-up schedules. It also explains why ADL meta-regression and subgroup analyses should be treated as exploratory.

The model should still be interpreted within the limits of the evidence. A three-level approach improves the handling of dependent effect sizes, but it does not increase the number of independent RCTs. The study-level evidence remains small. The model therefore improves the analysis of the available data, but it cannot remove uncertainty caused by few trials, small samples, and heterogeneous protocols.

### Clinical and research implications

The current evidence is insufficient to support routine implementation of adjunctive ESWT after BTX-A. The pooled data suggest a possible short-term benefit for impairment-level outcomes, particularly muscle tone and limb-level motor performance, but certainty is moderate for MAS and low for FMA, and reliable improvement in global functional independence has not been demonstrated. ESWT should therefore not be presented as an evidence-based means of restoring ADL independence on the basis of this review. No recommendation can be made regarding focused vs. radial ESWT, treatment dose, injection-to-ESWT timing, or anatomical target. If ESWT is used in practice, the decision should remain individualized and embedded within a broader rehabilitation plan rather than inferred from the pooled estimates as a routine standard of care.

This caution is consistent with recent broader evidence. A 2026 umbrella review concluded that ESWT signals are generally favorable but that the underlying evidence remains limited by methodological quality and certainty (33). Future trials should use BTX-A-based control conditions, preferably with sham ESWT when feasible, and should standardize injection guidance, dose, target muscles, ESWT modality, energy parameters, session number, and timing after injection. Assessor blinding and adverse-event reporting should be consistent. Trials should also provide enough data for both endpoint and change-score analyses. Direct comparisons of focused and radial ESWT are needed before modality-specific recommendations can be made.

Functional outcomes need particular care. Trials should state whether the main goal is tone reduction, limb-level motor recovery, or functional independence, because these outcomes are related but not interchangeable. If ADL is a primary target, focal treatment should be paired with standardized task-specific rehabilitation (27), and follow-up should be long enough to detect functional transfer. Otherwise, future studies may continue to show impairment-level benefits while leaving the functional impact unclear.

### Limitations

Several limitations should temper interpretation. First, the primary quantitative synthesis included only six independent RCTs and 242 participants. The three-level model appropriately handles dependent effect sizes but does not increase the number of independent trials. Second, intervention heterogeneity was substantial: trials differed in stroke chronicity, injected and shock-wave-treated muscles, BTX-A guidance and dose reporting, ESWT modality, pressure or EFD, impulse number, number of sessions, timing after injection, rehabilitation co-interventions, outcome instruments, and follow-up schedules. Third, one trial had high risk of bias in outcome measurement and other trials had varying limitations in reporting and blinding. Fourth, the primary effect-size metric used post-treatment standardized mean differences, which are vulnerable to baseline imbalance; this was particularly relevant to ADL and the single focused-ESWT trial. Fifth, fewer than 10 independent studies were available for any outcome, precluding reliable formal assessment of publication bias or small-study effects. Finally, trial-level toxin dose and ESWT exposure were not reported in sufficiently comparable formats to support a reliable dose-response or toxin-dose x ESWT-type interaction analysis. These limitations reduce certainty and restrict clinical generalizability despite the stability of the MAS and FMA direction in sensitivity analyses. Adverse-event ascertainment and reporting were inconsistent, so absence of reported events should not be interpreted as evidence of safety.

## Conclusion

In RCTs with BTX-A-based control conditions, adjunctive ESWT was associated with greater short-term reductions in post-stroke spasticity and greater improvements in motor impairment. Certainty was moderate for MAS and low for FMA, while evidence that these impairment-level gains translate into improved global functional independence was very uncertain. Findings related to ESWT modality, anatomical target, treatment intensity, toxin dose, and timing remain exploratory. Larger, adequately blinded trials with standardized BTX-A dosing, clearly specified target muscles, harmonized ESWT parameters, and prespecified functional outcomes are required before routine clinical recommendations or dose-response conclusions can be made. The comparative safety of adjunctive ESWT also remains uncertain.

## Data Availability

The data extracted for this review are included in the article/Supplementary Material; analysis files are available from the corresponding author on reasonable request.
