# Supplementary material for: Adjunctive extracorporeal shock wave therapy after botulinum toxin type A injection for post-stroke spasticity: a systematic review and three-level meta-analysis
Source: Front Neurol. 2026 Oct 20;17:1890975. doi: 10.3389/fneur.2026.1890975 (PMC13727453; doi:10.3389/fneur.2026.1890975)
Supplement: Supplementary file 1 [file Data_Sheet_1.pdf]

PRISMA 2020 Checklist — Revised

Manuscript ID 1890975 | Frontiers in Neurology

Section and Topic	Item	Checklist Item	Reported in Revised Manuscript
TITLE	1	Identify the report as a systematic review and/or meta-analysis.	Title page
ABSTRACT	2	See the PRISMA 2020 for Abstracts checklist.	Abstract
INTRODUCTION	3	Describe the rationale for the review in the context of existing knowledge.	Introduction
INTRODUCTION	4	Provide an explicit statement of the objective(s) or question(s) the review addresses.	Introduction
METHODS	5	Specify inclusion and exclusion criteria.	Methods: Selection criteria / PICOS framework
METHODS	6	Specify all databases and sources searched.	Methods: Search strategy
METHODS	7	Present full search strategies.	Supplementary Material: eTable 2
METHODS	8	Specify methods for study selection.	Methods: Study selection
METHODS	9	Specify methods for data extraction.	Methods: Data extraction and MAS transformation
METHODS	10a	Define all outcomes sought.	Methods: PICOS framework
METHODS	10b	Define all other variables sought.	Methods: Data extraction; Table 2; Supplementary eTables 3–4
METHODS	11	Specify risk-of-bias assessment methods.	Methods: Quality assessment
METHODS	12	Specify effect measures.	Methods: Statistical analysis
METHODS	13a–f	Describe synthesis methods and sensitivity analyses.	Methods: Statistical analysis
METHODS	14	Describe assessment of reporting bias.	Methods: Statistical analysis; Results: Sensitivity analysis and publication bias
METHODS	15	Describe certainty/confidence assessment.	Methods: Certainty of evidence
RESULTS	16a	Describe search and selection process.	Results: Study selection and characteristics; Figure 1
RESULTS	16b	List excluded studies and reasons.	Results: Study selection and characteristics
RESULTS	17	Present study characteristics.	Main Table 2; Supplementary eTables 3–4
RESULTS	18	Present risk-of-bias results.	Results: Methodological quality; Supplementary eFigures 1–2
RESULTS	19	Present individual study results.	Figure 3; Supplementary Material
RESULTS	20a–d	Present synthesis, heterogeneity, and sensitivity analyses.	Results: Primary/secondary outcomes; meta-regression; sensitivity analyses
RESULTS	21	Present reporting-bias assessment.	Results: Sensitivity analysis and publication bias
RESULTS	22	Present certainty of evidence.	Results: Certainty of evidence; Table 1 (Summary of Findings)
DISCUSSION	23a–d	Interpret findings, limitations, and implications.	Discussion
OTHER INFORMATION	24a	Provide registration information.	Abstract; Methods: Study protocol and registration
OTHER INFORMATION	24b	Provide protocol access information.	Methods: Study protocol and registration
OTHER INFORMATION	24c	Describe protocol amendments.	Methods: Study protocol and registration
OTHER INFORMATION	25	Describe funding/support.	Funding statement
OTHER INFORMATION	26	Declare competing interests.	Conflict of interest statement
OTHER INFORMATION	27	Describe data/material availability.	Data availability statement
