# Supplementary material for: Adjunctive extracorporeal shock wave therapy after botulinum toxin type A injection for post-stroke spasticity: a systematic review and three-level meta-analysis
Source: Front Neurol. 2026 Oct 20;17:1890975. doi: 10.3389/fneur.2026.1890975 (PMC13727453; doi:10.3389/fneur.2026.1890975)
Supplement: Supplementary file 2 [file Supplementary_file_1.docx]

**Supplementary Material**

**eTable 1: Definitions of outcomes**

**eTable 2: Search strategy**

*Note: Full search strategies are shown for PubMed, Embase, the Cochrane Library, Web of Science, and CBM. Platform-specific Chinese query strings for CNKI, Wanfang Data, and VIP are not reproduced here; these databases were searched using equivalent Chinese terms for stroke, spasticity, randomized trials, BTX-A, and ESWT.*

**eTable 3: Characteristics of trials included in the primary quantitative synthesis.**

eTable 4: Detailed BTX-A and ESWT intervention protocols across included trials

**eTable 5: Sensitivity analyses of primary outcomes.**

**eTable 6: Exploratory univariate meta-regression analyses.**

**eTable 7: Study-clustered CR2 sensitivity analyses.**

**eFigure 1: Risk of bias summary for trials contributing to the primary quantitative synthesis.**

**eFigure 2: Risk of bias graph across trials contributing to the primary quantitative synthesis.**

**Supplementary Method 1: Mathematical Formulation of the Three-Level Meta-Analysis Model**

**eTable 1: Definitions of outcomes**

| **Outcomes** | **Definitions** |
| --- | --- |
| MAS | Modified Ashworth Scale: A clinical tool used to assess muscle tone. For this meta-analysis, ordinal scores (0, 1, 1+, 2, 3, 4) were transformed into a continuous 6-point scale (0, 1, 1.5, 2, 3, 4). |
| FMA | Fugl-Meyer Assessment: A stroke-specific performance-based measure. The included trials used upper-extremity, lower-extremity, or total motor scores depending on the treated population. |
| ADL | Activities of Daily Living: Global functional independence. In the included trials, ADL was measured with the Barthel Index (BI) or Modified Barthel Index (MBI); these are not upper- or lower-limb-specific scales. |

**eTable 2: Search strategy**

| **PubMed** | | |
| --- | --- | --- |
| No. | Query | Results |
| 1 | "Randomized Controlled Trial"[Publication Type] OR "Randomized Controlled Trials as Topic"[Mesh] OR Randomized[tiab] OR Randomly[tiab] OR Controlled*[tiab] OR Trial*[tiab] OR RCT[tiab] OR RCTs[tiab] | 2,917,161 |
| 2 | "Stroke"[Mesh] OR Stroke[tiab] OR "Cerebrovascular Accident"[tiab] OR "Cerebral Stroke*"[tiab] OR "Cerebrovascular Apoplex*"[tiab] OR "Brain Vascular Accident*"[tiab] OR "Cerebrovascular Stroke*"[tiab] OR Apoplex*[tiab] OR CVA[tiab] OR CVAs[tiab] | 425,960 |
| 3 | "Muscle Spasticity"[Mesh] OR Spastic[tiab] OR Muscle Spasticit[tiab] OR Clasp-Knife*[tiab] OR Clasp Knife*[tiab] | 28,054 |
| 4 | "Extracorporeal Shockwave Therapy"[Mesh] OR "High-Intensity Focused Ultrasound Ablation"[Mesh] OR Extracorporeal Shockwave Therap[tiab] OR Extracorporeal Shock Wave Therap[tiab] OR Shock Wave Therap*[tiab] OR ESWT[tiab] OR rESWT[tiab] OR fESWT[tiab] OR HIFU*[tiab] OR High Intensity Focused Ultrasound Therap*[tiab] | 8,470 |
| 5 | "Botulinum Toxins, Type A"[Mesh] OR Botulinum Toxin[tiab] OR Botulinum* Neurotoxin*[tiab] OR Clostridium botulinum*[tiab] OR Oculinum*[tiab] OR Botox*[tiab] OR Meditoxin*[tiab] OR Vistabel*[tiab] OR Neuronox*[tiab] OR Vistabex*[tiab] OR BTX-A[tiab] OR BTXA[tiab] | 28,297 |
| 6 | #1 AND #2 AND #3 AND #4 AND #5 | 8 |
| **Embase** | | |

| No. | Query | Results |
| --- | --- | --- |
| #1 | 'cerebrovascular accident'/exp | 547,560 |
| #2 | 'stroke':ti,ab OR 'strokes':ti,ab OR 'cerebrovascular accident':ti,ab OR 'cerebral stroke':ti,ab OR 'apoplexy':ti,ab OR 'cva':ti,ab | 637,889 |
| #3 | #1 OR #2 | 770,638 |
| #4 | 'spasticity'/exp | 44,889 |
| #5 | 'spasticity':ti,ab OR 'spastic':ti,ab OR 'muscle spasticity':ti,ab OR 'muscle hypertonia':ti,ab OR 'clasp-knife':ti,ab | 51,460 |
| #6 | #4 OR #5 | 70,374 |
| #7 | 'extracorporeal shock wave therapy'/exp | 4,919 |
| #8 | 'extracorporeal shockwave therapy':ti,ab OR 'shock wave therapy':ti,ab OR 'eswt':ti,ab OR 'reswt':ti,ab OR 'feswt':ti,ab | 5,458 |
| #9 | #7 OR #8 | 7,025 |
| #10 | 'botulinum toxin type a'/exp | 33,017 |
| #11 | 'botulinum toxin type a':ti,ab OR 'botox':ti,ab OR 'dysport':ti,ab OR 'xeomin':ti,ab OR 'btx a':ti,ab OR 'btxa':ti,ab | 15,110 |
| #12 | #10 OR #11 | 34,216 |
| #13 | 'randomized controlled trial'/exp OR 'randomization'/exp OR 'controlled clinical trial'/exp OR 'randomized':ti,ab OR 'trial':ti,ab OR 'rct':ti,ab | 6,588,399 |
| #14 | #3 AND #6 AND #9 AND #12 AND #13 | 16 |
| **Cochrane database** | | |
| No. | Query | Results |
| #1 | MeSH descriptor: [Stroke] explode all trees | 18,941 |
| #2 | (stroke OR strokes OR "cerebrovascular accident" OR "cerebral stroke" OR apoplexy OR cva):ti,ab,kw | 84,720 |
| #3 | #1 OR #2 | 85,258 |
| #4 | MeSH descriptor: [Muscle Spasticity] explode all trees | 1,374 |
| #5 | (spasticity OR spastic OR "muscle spasticity" OR "muscle hypertonia" OR "clasp-knife"):ti,ab,kw | 6,562 |
| #6 | #4 OR #5 | 6,562 |
| #7 | MeSH descriptor: [Extracorporeal Shockwave Therapy] explode all trees | 369 |
| #8 | ("extracorporeal shockwave therapy" OR "shock wave therapy" OR eswt OR reswt OR feswt):ti,ab,kw | 5,458 |
| #9 | #7 OR #8 | 5,458 |
| #10 | MeSH descriptor: [Botulinum Toxins, Type A] explode all trees | 957 |
| #11 | #3 AND #6 AND #9 | 504 |
| #12 | #11 AND #10 | 9 |
| **Web of Science** | | |
| No. | Query | Results |
| 1 | TS=("Stroke" OR "Strokes" OR "Cerebrovascular Accident" OR "Cerebral Stroke" OR "Cerebrovascular Apoplexy" OR "Brain Vascular Accident*" OR "CVA" OR "CVAs") | 538,507 |
| 2 | TS=("Spasticity" OR "Spasticities" OR "Spastic" OR "Muscle Spasticity" OR "Muscle Spasticities" OR "Muscle Hypertonia" OR "Clasp-knife" OR "Clasp knife") | 35,564 |
| 3 | TS=("Extracorporeal Shockwave Therapy" OR "Extracorporeal Shock Wave Therapy" OR "Shock Wave Therapy" OR "Shockwave Therapy" OR "ESWT" OR "rESWT" OR "fESWT") | 4,952 |
| 4 | TS=("Botulinum Toxin Type A" OR "Botulinum Toxin A" OR "Botulinum A Toxin" OR "Botulinum Neurotoxin A" OR "Botox" OR "Dysport" OR "Xeomin" OR "Prosigne" OR "Meditoxin" OR "Neuronox" OR "BTX A" OR "BTXA") | 14,196 |
| 5 | TS=("Randomized Controlled Trial" OR "Randomized" OR "Randomised" OR "Randomly" OR "Trial" OR "RCT" OR "RCTs") | 2,580,122 |
| 6 | #5 AND #4 AND #3 AND #2 AND #1 | 10 |
| **CBM database** | | |
| No. | Query | Results |
| 1 | 脑卒中 OR 中风 OR 偏瘫 OR 脑梗 OR 脑出血 OR 脑血管意外 | 1,002,689 |
| 2 | 痉挛 OR 肌张力 OR 改良Ashworth OR Ashworth评分 | 157,490 |
| 3 | 随机 OR 对照 OR 随机分配 OR RCT | 4,204,289 |
| 4 | 肉毒毒素 OR A型肉毒毒素 OR 衡力 OR 保妥适 | 12,631 |
| 5 | 冲击波 OR 体外冲击波 | 14,359 |
| 6 | (1) AND (2) AND (3) AND (4) AND (5) | 7 |

**eTable 3: Characteristics of trials included in the primary quantitative synthesis.**

| **Study (Year)** | **Sample Size (E / C1 / C2)** | **Age (Years, Mean ± SD)** | **Stroke Stage** | **Limb Location** | **Control Group Content** | **Outcomes** |
| --- | --- | --- | --- | --- | --- | --- |
| Talay Çalış et al. (2026; online 2025) | 20 / 20 | E: 62.5 ± 9.23 C: 57.4 ± 10.17 | Chronic | Lower limb | BTX-A + Conventional Rehab | MAS, BI, ROM, VAS, CS |
| Deng (2026) | 25 / 25 | Range: 40–65 | Chronic / Subacute | Upper limb | BTX-A + Conventional Rehab | MAS, FMA, MBI |
| Duan (2020) | 18 / 18 | E: 51.8 ± 6.20 C: 52.6 ± 7.90 | Subacute | Lower limb | BTX-A + Sham-ESWT | MAS, FMA, MBI, PROM |
| Lee (2024) | 9 / 7 | E: 59.0 (37–62.5) C: 52.0 (42–62) | Chronic | Upper limb | BTX-A alone | MAS, FMA, MBI, MTS, ARAT |
| Ren (2025) | 30 / 30 | E: 67.5 ± 3.40 C: 67.9 ± 3.30 | Subacute | Limb spasticity (Upper & Lower) | BTX-A + Conventional Rehab | MAS, FMA, MBI, VAS |
| Wang (2017) | 20 / 20 | E: 52.2 ± 13.7 C: 50.9 ± 15.2 | Chronic | Lower limb | BTX-A + Conventional Rehab | MAS, FMA, MBI |

Notes: Abbreviations: E = experimental arm (ESWT + BTX-A); C / Ctrl = BTX-A-based control arm included in the primary quantitative meta-analysis. Only comparison arms with equivalent BTX-A-based control conditions were included in the primary quantitative synthesis to preserve the add-on estimand. The primary quantitative synthesis included 6 RCTs and 242 participants. Liang et al. (2021) was retained for qualitative review but excluded from the primary quantitative synthesis because its comparator did not include a BTX-A-based baseline.

Participant denominators: The seven RCTs reported analytic populations totaling 377 participants (Talay Çalış, 40; Deng, 75; Duan, 36; Lee, 16; Liang, 60; Ren, 90; Wang, 60). Of these, 242 participants contributed to eligible BTX-A-based contrasts. The other 135 participants were from Liang et al. (n = 60) and non-BTX-A comparator arms in Deng (n = 25), Ren (n = 30), and Wang (n = 20). Talay Çalış et al. randomized 47 participants; 40 completed follow-up and were analyzed.

**eTable 3b: Trial retained for qualitative synthesis but excluded from the primary quantitative synthesis.**

| **Study (Year)** | **Trial N** | **Intervention** | **Comparator** | **Reason excluded from primary quantitative synthesis** |
| --- | --- | --- | --- | --- |
| Liang et al. (2021) | 60 | BTX-A + ESWT + conventional rehabilitation | Conventional rehabilitation without BTX-A baseline | Lacked equivalent BTX-A-based comparator; retained for qualitative review only |

eTable 4: Detailed BTX-A and ESWT intervention protocols across included trials

| **Study** | **Status** | **Limb / target pattern** | **BTX-A: guidance, muscles, dose** | **ESWT: modality / treated muscles / parameters** | **Sessions / timing after BTX-A** | **Functional outcomes** |
| --- | --- | --- | --- | --- | --- | --- |
| Talay Çalış et al. (2026; online 2025) | Quantitative | Lower; chronic ankle plantar-flexor spasticity | US-guided gastrocnemius; medial head 50 IU + lateral head 25 IU | Radial; medial/lateral gastrocnemius; 0.1 mJ/mm² and 2 bar; 4 Hz; 1000 impulses | 4 sessions (2/week); starts day 7 | MAS + global BI |
| Deng (2026) | Quantitative | Upper; elbow/wrist/finger flexor spasticity | US-guided individualized target muscles; Botox 100 U/2 mL preparation; exact total dose and named muscles not reported | Radial; individualized spastic target-muscle bellies; 1.8–2.5 bar; 8–10 Hz; 2000 impulses | 24 sessions (2/week × 12 weeks); starts day 3 (~48 h) | MAS; upper-extremity FMA; global MBI |
| Duan et al. (2020) | Quantitative | Lower; subacute triceps surae | Electrical-stimulation guidance; gastrocnemius medial/lateral heads + soleus; individualized; maximum 100 U per muscle group | Radial; triceps surae; 2.0–3.0 bar; 8 Hz; 2000 impulses | Regimen: daily on post-injection days 1–3, then every 3 days from week 2 through week 4; starts 24 h after BTX-A | MAS; lower-extremity FMA; global MBI |
| Lee & Yang (2024) | Quantitative | Upper; chronic elbow/wrist/finger flexors | Electrical-stimulation guidance; individualized biceps brachii, brachioradialis, pronator teres, FCR, FCU, FDP, FDS; muscle-specific doses reported | Focused; middle belly of biceps brachii or FCR; EFD 0.030 mJ/mm²; 4 Hz; 1000 shots | 5 daily sessions; begins immediately | MAS/MTS; upper-extremity FMA; ARAT; global MBI |
| Ren & Hu (2025) | Quantitative | Both upper and lower limbs; subacute unilateral spasticity | US-guided individualized targets; 2–4 sites/muscle, 10–25 U/site; maximum ≤600 U per treated side; exact named targets not listed | Radial; target muscle belly; 2 bar; 6 Hz; EFD 0.03–0.24 mJ/mm²; 2000 impulses/muscle group | 4 weekly sessions; starts day 3 (~48 h) | Affected-limb MAS; total FMA (UE+LE); global MBI |
| Wang et al. (2017) | Quantitative | Lower; chronic ankle plantar-flexor/triceps-surae pattern | EMG guidance; candidates included gastrocnemius, soleus, tibialis posterior, FHL, FDL; individualized; total 400 U | Radial; gastrocnemius + soleus; 2 bar; 8 Hz; 1500–2000 impulses | 4 weekly sessions; starts next day (~24 h) | MAS of ankle plantar flexors; lower-extremity FMA; global MBI |
| Liang et al. (2021) | Qualitative only | Upper limb | US-guided individualized visibly spastic target muscles; Botox 100 U/2 mL preparation; exact named muscles/total dose not reported | Modality not explicitly labeled in the available report; shock wave applied to the spastic muscle belly; 2 bar; 10 Hz; 2000–3000 impulses | 12 sessions (2/week × 6 weeks); starts day 2 | Upper-limb MAS/FMA; global MBI |

Notes: BI/MBI are global ADL instruments. Liang et al. (2021) is included for qualitative completeness but was excluded from the quantitative add-on model because its comparator lacked a BTX-A baseline. Radial pressure (bar) and focused EFD (mJ/mm²) are not directly equivalent dose units. Abbreviations: ADL, activities of daily living; ARAT, Action Research Arm Test; BI, Barthel Index; BTX-A, botulinum toxin type A; EFD, energy flux density; EMG, electromyography; ESWT, extracorporeal shock wave therapy; FCR/FCU, flexor carpi radialis/ulnaris; FDP/FDS, flexor digitorum profundus/superficialis; FHL/FDL, flexor hallucis/digitorum longus; FMA, Fugl-Meyer Assessment; MAS, Modified Ashworth Scale; MBI, Modified Barthel Index; MTS, Modified Tardieu Scale; US, ultrasound.

Supplementary safety summary

Safety findings: Safety outcomes were reported inconsistently. Talay Çalış et al. reported transient erythema in 2 of 20 adjunctive-ESWT participants, lasting about 20 minutes, with no other side effects. Duan et al. reported no obvious adverse reactions, and Lee and Yang reported no serious adverse effects or complications. Liang et al. reported no allergic reactions after BTX-A but did not otherwise summarize ESWT-related adverse events. Deng et al., Ren and Hu, and Wang et al. did not provide usable trial-level safety data. These data did not support a comparative safety estimate.

**eFigure 1: Risk of bias summary for trials contributing to the primary quantitative synthesis.**

**Risk-of-bias judgments are shown for each domain and for each included study.**

**eFigure 2: Risk of bias graph across trials contributing to the primary quantitative synthesis.**

**Risk-of-bias judgments are presented as percentages across all included studies.**

**Supplementary Method 1: Mathematical Formulation of the Three-Level Meta-Analysis Model**

To address dependency among effect sizes extracted from the same trial, let y_ij denote the j-th effect size from the i-th study. The three-level random-effects model partitions variability into sampling error, within-study heterogeneity, and between-study heterogeneity.

**Level 1 (Sampling Error):**

y_ij = θ_ij + ε_ij

where θ_ij is the true effect for the j-th effect size in study i and ε_ij is the sampling error. We assume ε_ij ~ N(0, v_ij), where v_ij is the known sampling variance.

**Level 2 (Within-Study Variance):**

θ_ij = κ_i + ζ_ij

where κ_i is the average true effect for study i and ζ_ij is the within-study deviation for effect size j. We assume ζ_ij ~ N(0, τ²₂), where τ²₂ is the within-study variance component.

**Level 3 (Between-Study Variance):**

κ_i = μ + η_i

where μ is the overall mean true effect and η_i is the deviation of study i from that mean. We assume η_i ~ N(0, τ²₃), where τ²₃ is the between-study variance component.

**Combined Full Model:**

Substituting the components, the full three-level structural equation is:

y_ij = μ + η_i + ζ_ij + ε_ij

Under the model assumptions, Var(y_ij) = v_ij + τ²₂ + τ²₃. This structure allows dependent effect sizes from the same trial to be retained without treating them as independent observations.

Analyses were conducted in R. The initial models used R v4.3.1 and metafor v4.4-0; the revised FMA and CR2 analyses used R v4.6.0, metafor v5.0-1, and clubSandwich v0.7.0.

**eTable 5. Sensitivity analyses of primary outcomes.**

| **Outcome** | **Sensitivity model** | **k** | **m** | **Hedges g** | **95% CI** | **P value** |
| --- | --- | --- | --- | --- | --- | --- |
| MAS / spasticity | Main post-treatment SMD model | 6 | 21 | 0.87 | 0.41 to 1.34 | <0.001 |
| MAS / spasticity | Exclude Ren 2025 (high risk in outcome measurement) | 5 | 17 | 0.74 | 0.25 to 1.23 | 0.003 |
| MAS / spasticity | Follow-up closest to week 4 only | 6 | 8 | 0.76 | 0.41 to 1.12 | <0.001 |
| MAS / spasticity | Study-level aggregation at closest week 4 | 6 | 6 | 0.73 | 0.39 to 1.07 | <0.001 |
| FMA / motor function | Main post-treatment SMD model | 5 | 14 | 1.11 | 0.45 to 1.76 | <0.001 |
| FMA / motor function | Exclude Ren 2025 (high risk in outcome measurement) | 4 | 10 | 0.77 | 0.46 to 1.09 | <0.001 |
| FMA / motor function | Follow-up closest to week 4 only | 5 | 5 | 1.33 | 0.58 to 2.08 | <0.001 |
| FMA / motor function | Study-level aggregation at closest week 4 | 5 | 5 | 1.33 | 0.58 to 2.08 | <0.001 |
| ADL | Main post-treatment SMD model | 6 | 17 | 1.15 | -0.03 to 2.32 | 0.056 |
| ADL | Exclude Ren 2025 (high risk in outcome measurement) | 5 | 13 | 1.12 | -0.36 to 2.61 | 0.138 |
| ADL | Follow-up closest to week 4 only | 6 | 6 | 1.31 | 0.05 to 2.58 | 0.042 |
| ADL | Study-level aggregation at closest week 4 | 6 | 6 | 1.31 | 0.05 to 2.58 | 0.042 |
| ADL | Exclude Lee 2024 (focused ESWT) | 5 | 15 | 0.62 | 0.11 to 1.13 | 0.018 |
| ADL | Exclude major baseline imbalance | 5 | 15 | 0.62 | 0.11 to 1.13 | 0.018 |
| ADL | ADL: MBI only | 5 | 15 | 1.44 | 0.15 to 2.73 | 0.029 |
| ADL | ADL: BI only | 1 | 2 | -0.26 | -0.70 to 0.18 | 0.242 |
| ADL | Change-score SMD, r = 0.2 | 6 | 17 | 0.41 | -0.07 to 0.89 | 0.091 |
| ADL | Change-score SMD, r = 0.5 | 6 | 17 | 0.49 | -0.13 to 1.10 | 0.119 |
| ADL | Change-score SMD, r = 0.8 | 6 | 17 | 0.70 | -0.23 to 1.63 | 0.140 |

Notes: Positive Hedges g values favor ESWT + BTX-A. Change-score SMD models for ADL were calculated under assumed pre-post correlations (r) of 0.2, 0.5, and 0.8. k = number of independent studies; m = number of effect sizes.

**eTable 6. Exploratory univariate meta-regression analyses.**

| **Outcome** | **Moderator** | **Type** | **k** | **m** | **QM** | **df** | **P value** |
| --- | --- | --- | --- | --- | --- | --- | --- |
| MAS / spasticity | ESWT modality | categorical | 6 | 21 | 3.80 | 1 | 0.051 |
| MAS / spasticity | Limb subgroup | categorical | 5 | 17 | 0.02 | 1 | 0.875 |
| MAS / spasticity | Follow-up period | categorical | 6 | 21 | 1.27 | 2 | 0.530 |
| MAS / spasticity | Time point (weeks) | continuous | 6 | 21 | 0.06 | 1 | 0.802 |
| MAS / spasticity | Total ESWT sessions | continuous | 6 | 21 | 0.32 | 1 | 0.571 |
| MAS / spasticity | Days from BTX-A to ESWT | continuous | 6 | 21 | 0.59 | 1 | 0.444 |
| MAS / spasticity | Stroke stage (months) | continuous | 6 | 21 | 4.53 | 1 | 0.033 |
| FMA / motor function | ESWT modality | categorical | 5 | 14 | 0.05 | 1 | 0.832 |
| FMA / motor function | Limb subgroup | categorical | 4 | 10 | 0.12 | 1 | 0.733 |
| FMA / motor function | Follow-up period | categorical | 5 | 14 | 1.35 | 2 | 0.510 |
| FMA / motor function | Time point (weeks) | continuous | 5 | 14 | 0.13 | 1 | 0.719 |
| FMA / motor function | Total ESWT sessions | continuous | 5 | 14 | 0.92 | 1 | 0.338 |
| FMA / motor function | Days from BTX-A to ESWT | continuous | 5 | 14 | 0.26 | 1 | 0.613 |
| ADL | ESWT modality | categorical | 6 | 17 | 16.94 | 1 | <0.001 |
| ADL | Limb subgroup | categorical | 5 | 13 | 2.28 | 1 | 0.131 |
| ADL | Follow-up period | categorical | 6 | 17 | 0.40 | 2 | 0.819 |
| ADL | Time point (weeks) | continuous | 6 | 17 | 0.30 | 1 | 0.583 |
| ADL | Total ESWT sessions | continuous | 6 | 17 | 0.11 | 1 | 0.740 |
| ADL | Days from BTX-A to ESWT | continuous | 6 | 17 | 2.57 | 1 | 0.109 |
| ADL | Stroke stage (months) | continuous | 6 | 17 | 7.59 | 1 | 0.006 |
| ADL excluding Lee 2024 | Limb subgroup | categorical sensitivity | 4 | 11 | 0.53 | 1 | 0.468 |
| ADL excluding Lee 2024 | Follow-up period | categorical sensitivity | 5 | 15 | 0.44 | 2 | 0.803 |
| ADL excluding major baseline imbalance | Limb subgroup | categorical sensitivity | 4 | 11 | 0.53 | 1 | 0.468 |
| ADL excluding major baseline imbalance | Follow-up period | categorical sensitivity | 5 | 15 | 0.44 | 2 | 0.803 |

Notes: Meta-regression analyses were univariate and exploratory. Categorical moderators are reported as omnibus QM tests. For FMA, the Lee follow-up was coded at 12 weeks, consistent with the source table, and the miscoded baseline row was removed. The FMA stroke-stage model was not retained because stroke duration was not reported comparably across the five trials. Moderator findings should not be interpreted as confirmatory evidence of effect modification.

**eTable 7. Study-clustered CR2 sensitivity analyses.**

| **Outcome** | **CR2 analysis** | **k** | **m** | **Hedges g** | **CR2 95% CI** | **df** | **P value** |
| --- | --- | --- | --- | --- | --- | --- | --- |
| MAS | Primary post-treatment SMD | 6 | 21 | 0.87 | 0.25 to 1.49 | 4.79 | 0.016 |
| FMA | Primary post-treatment SMD | 5 | 14 | 1.11 | 0.16 to 2.05 | 3.84 | 0.032 |
| ADL | Primary post-treatment SMD | 6 | 17 | 1.15 | -0.39 to 2.68 | 4.97 | 0.112 |
| FMA | Omit Deng 2026 | 4 | 10 | 1.23 | -0.17 to 2.62 | 2.86 | 0.068 |
| FMA | Omit Duan 2020 | 4 | 12 | 1.31 | 0.13 to 2.49 | 2.80 | 0.039 |
| FMA | Omit Lee 2024 | 4 | 13 | 1.07 | -0.17 to 2.32 | 2.99 | 0.071 |
| FMA | Omit Ren 2025 | 4 | 10 | 0.77 | 0.19 to 1.36 | 2.11 | 0.028 |
| FMA | Omit Wang 2017 | 4 | 11 | 1.11 | -0.25 to 2.48 | 2.93 | 0.081 |

Notes: CR2 estimates were clustered by study and used Satterthwaite small-sample degrees of freedom. Point estimates are unchanged from the corresponding three-level models. k = number of independent studies; m = number of effect sizes.
